# Supplementary material for: An Experimental Study Evaluating the Influence of Front-of-Package Warning Labels on Adolescent’s Purchase Intention of Processed Food Products
Source: Int J Environ Res Public Health. 2022 Jan 19;19(3):1094. doi: 10.3390/ijerph19031094 (PMC8833989; doi:10.3390/ijerph19031094)
Supplement: Supplementary file 1 [file ijerph-19-01094-s001.zip › ijerph-1477692-supplementary.pdf]

International Journal of
Environmental Research and Public Health

MDPI

Supplementary Material

Table S1. Nutritional composition per 100g of the six processed products included in the experiment.

Product	Sugar (g)	Saturated fats (g)	Sodium (mg)
**Cookies**			
Vainilla cookies (0 WL)	21.6	4.1	405.4
Plain cookies (1 WL)	21.5	7.5*	408.6
Chocolate filled cookies (2 WL)	30.6*	9.7*	333.3
**Salty Snacks**			
Pop corn (0 WL)	1.0	2.5	480.0
Fried tortillas (1 WL)	4.0	12.0*	710.0
Puffed corn snacks (2 WL)	1.0	11.0*	1160.0*

WL: Warning labels, * Product "high-in" critical nutrient according to the Supreme Decree N° 012-2018-SA.

Table S2. Differences in intention to purchase by school.

	School 1			School 2		
	**Control*** n (%)	**Experimental** n(%)	p	**Control*** n (%)	**Experimental** n(%)	p
**Cookies**			0.242			0.357
Vainilla cookies (0 WL)	36 (54.6)	41 (54.7)		31 (40.8)	24 (31.6)	
Plain cookies (1 WL)	17 (25.8)	26 (34.7)		24 (31.6)	32 (42.1)	
Chocolate filled cookies (2 WL)	13 (19.7)	8 (10.6)		21 (27.6)	20 (26.3)	
**Salty Snacks**			0.163			0.440
Pop corn (0 WL)	14 (37.8)	17 (58.6)		24 (53.3)	19 (42.2)	
Fried tortillas (1 WL)	17 (46.0)	7 (24.1)		16 (35.6)	22 (48.9)	
Puffed corn snacks (2 WL)	6 (16.2)	5 (17.3)		5 (11.1)	4 (8.9)	
**All**			0.422			0.162
Vainilla cookies and pop corn (0 WL)	50 (48.5)	58 (55.8)		55 (45.5)	43 (35.5)	
Plain cookies and fried tortillas (1 WL)	34 (33.0)	33 (31.7)		40 (33.1)	54 (44.6)	
Chocolate filled cookies and puffed corn snack (2 WL)	19 (18.5)	13 (12.5)		26 (21.5)	24 (19.8)	

* Packaged food without warning labels, WL: Warning labels

Table S3. Differences in identification of healthiest food by school.

	School 1			School 2		
	**Control*** n (%)	**Experimental** n(%)	p	**Control*** n (%)	**Experimental** n(%)	p
**Cookies**			0.498			0.149
Vainilla cookies (0 WL)	58 (87.9)	61 (81.3)		57 (75.0)	65 (85.5)	
Plain cookies (1 WL)	7 (10.6)	11 (14.7)		17 (22.4)	11 (14.5)	
Chocolate filled cookies (2 WL)	1 (1.5)	3 (4.0)		2 (2.6)	0 (0.0)	
**Salty Snacks**			0.231			0.365
Pop corn (0 WL)	34 (91.9)	24 (82.8)		39 (86.7)	38 (84.4)	
Fried tortillas (1 WL)	2 (5.4)	1 (3.5)		3 (6.7)	1 (2.2)	
Puffed corn snacks (2 WL)	1 (2.7)	4 (13.8)		3 (6.6)	6 (13.3)	
**All**			0.176			0.311
Vainilla cookies and pop corn (0 WL)	92 (89.3)	85 (81.7)		96 (79.3)	103 (85.1)	
Plain cookies and fried tortillas (1 WL)	9 (8.7)	11 (11.5)		20 (16.5)	12 (9.9)	
Chocolate filled cookies and puffed corn snack (2 WL)	2 (1.9)	7 (6.7)		5 (4.2)	6 (5.0)	

* Packaged food without warning labels, WL: Warning labels

Table S4. Differences in intention to purchase by school level.

	Primary			Secondary		
	**Control*** n (%)	**Experimental** n(%)	p	**Control*** n (%)	**Experimental** n(%)	p
**Cookies**			0.380			0.017
Vainilla cookies (0 WL)	32 (57.3)	24 (45.3)		35 (42.7)	41 (41.8)	
Plain cookies (1 WL)	22 (33.7)	19 (39.8)		19 (23.2)	39 (39.8)	
Chocolate filled cookies (2 WL)	6 (10.0)	10 (18.9)		28 (34.1)	18 (18.4)	
**Salty Snacks**			0.711			0.925
Pop corn (0 WL)	9 (39.1)	16 (47.1)		29 (49.2)	20 (50.0)	
Fried tortillas (1 WL)	12 (57.2)	14 (41.2)		21 (35.6)	15 (37.5)	
Puffed corn snacks (2 WL)	2 (8.7)	4 (11.8)		9 (15.3)	5 (12.5)	
**All**			0.456			0.067
Vainilla cookies and pop corn (0 WL)	41 (49.4)	40 (46.0)		64 (45.4)	61 (44.2)	
Plain cookies and fried tortillas (1 WL)	34 (41.0)	33 (37.9)		40 (28.4)	54 (39.1)	
Chocolate filled cookies and puffed corn snack (2 WL)	8 (9.6)	14 (16.1)		37 (26.2)	23 (16.7)	

* Packaged food without warning labels, WL: Warning labels

Table S5. Differences in identification of healthiest food by school level.

		Primary			Secondary		
		**Control*** n (%)	**Experimental** n(%)	p	**Control*** n (%)	**Experimental** n(%)	p
**Cookies**				0.688			0.911
	Vainilla cookies (0 WL)	46 (76.7)	44 (83.0)		69 (84.2)	82 (83.7)	
	Plain cookies (1 WL)	12 (20.0)	8 (15.1)		12 (14.6)	14 (14.3)	
	Chocolate filled cookies (2 WL)	2 (3.3)	1 (1.9)		1 (1.2)	2 (2.0)	
	**Salty Snacks**			0.348			0.067
	Pop corn (0 WL)	20 (87.0)	25 (73.5)		53 (89.8)	37 (92.5)	
	Fried tortillas (1 WL)	0 (0.0)	2 (5.9)		5 (8.5)	0 (0.0)	
	Puffed corn snacks (2 WL)	3 (13.0)	7 (20.6)		1 (1.7)	3 (7.5)	
**All**				0.655			0.454
	Vainilla cookies and pop corn (0 WL)	66 (79.5)	69 (79.3)		122 (86.5)	119 (86.2)	
	Plain cookies and fried tortillas (1 WL)	12 (14.5)	10 (11.5)		17 (12.1)	14 (10.2)	
	Chocolate filled cookies and puffed corn snack (2 WL)	5 (6.0)	8 (9.2)		2 (1.4)	5 (3.6)	

* Packaged food without warning labels, WL: Warning labels

Table S6. Differences in intention to purchase by sex.

		Male			Female		
		**Control*** n (%)	**Experimental** n(%)	p	**Control*** n (%)	**Experimental** n(%)	p
**Cookies**				0.235			0.322
	Vainilla cookies (0 WL)	22 (40.7)	27 (45.0)		45 (51.1)	38 (41.8)	
	Plain cookies (1 WL)	17 (31.5)	24 (40.0)		24 (27.3)	34 (37.4)	
	Chocolate filled cookies (2 WL)	15 (27.8)	9 (15.0)		19 (21.6)	19 (20.8)	
	**Salty Snacks**			0.119			0.130
	Pop corn (0 WL)	17 (48.6)	19 (52.8)		21 (44.7)	17 (44,7)	
	Fried tortillas (1 WL)	16 (45.7)	10 (27.8)		17 (36.2)	19 (50.0)	
	Puffed corn snacks (2 WL)	2 (5.7)	7 (19.4)		9 (19.2)	2 (5.3)	
**All**				0.836			0.183
	Vainilla cookies and pop corn (0 WL)	39 (43.8)	46 (47.9)		66 (48.9)	55 (42.6)	
	Plain cookies and fried tortillas (1 WL)	33 (37.1)	34 (35.4)		41 (30.4)	53 (41.1)	
	Chocolate filled cookies and puffed corn snack (2 WL)	17 (19.1)	16 (16.7)		28 (20.7)	21 (16.3)	

* Packaged food without warning labels, WL: Warning labels

Table S7. Differences in identification of healthiest food by sex.

		Male			Female		
		**Control*** n (%)	**Experimental** n(%)	p	**Control*** n (%)	**Experimental** n(%)	p
**Cookies**				0.368			0.376
	Vainilla cookies (0 WL)	41 (75.9)	51 (85.0)		74 (84.1)	75 (82.4)	
	Plain cookies (1 WL)	10 (18.5)	8 (13.3)		14 (15.9)	14 (15.4)	
	Chocolate filled cookies (2 WL)	3 (5.6)	1 (1.7)		0 (0.0)	2 (2.2)	
	**Salty Snacks**			0.248			0.357
	Pop corn (0 WL)	31 (88.6)	31 (86.1)		42 (89.4)	31 (81.6)	
	Fried tortillas (1 WL)	3 (8.6)	1 (2.8)		2 (4.3)	1 (2.6)	
	Puffed corn snacks (2 WL)	1 (2.9)	4 (11.1)		3 (6.3)	6 (15.8)	
**All**				0.542			0.270
	Vainilla cookies and pop corn (0 WL)	72 (80.9)	82 (85.4)		116 (85.9)	106 (82.2)	
	Plain cookies and fried tortillas (1 WL)	13 (14.6)	9 (9.4)		16 (11.9)	15 (11.6)	
	Chocolate filled cookies and puffed corn snack (2 WL)	4 (4.5)	5 (5.2)		3 (2.2)	8 (6.2)	

* Packaged food without warning labels, WL: Warning labels

Table S8. Differences by number of warning labels between the experimental and control group.

**Outcome**		**β**	**se**	**p**	**95%CI**	
**Intention to purchase**	0 vs 2 WL	0.157	0.262	0.549	-0.357	0.671
	1 vs 2 WL	0.358	0.273	0.189	-0.177	0.892
**Identification of healthiest food**	0 vs 2 WL	-0.619	0.480	0.197	-1.560	0.322
	1 vs 2 WL	-0.808	0.544	0.137	-1.874	0.258

WL: Warning labels.
